# Daily Job Crafting Helps Those Who Help Themselves More: The Moderating Role of Job Autonomy and Leader Support

**DOI:** 10.3390/ijerph17062045

**Published:** 2020-03-19

**Authors:** Sung Hyoun Hong, Nayoung Kwon, Min Soo Kim

**Affiliations:** School of Business, Hanyang University, Seoul 04763, Korea; gener09@naver.com (S.H.H.); kny1030@hanyang.ac.kr (N.K.)

**Keywords:** daily job crafting, daily job satisfaction, job autonomy, leader support, multilevel analysis

## Abstract

Not all members are engaged in job crafting behavior in the same context, yet little research has addressed boundary conditions of daily job crafting. This study addresses these important issues and how the effects of daily job crafting vary depending on the work situation. We consider job autonomy and leader support as between-person level moderators and reveal how it affects the impact of daily job crafting on daily job satisfaction. Through the experience of the sampling method, we collected 946 days of data from 108 members (61.9% were male and 38.1% were female) for hypothesis testing. The analysis of results showed that the main effect of daily job crafting and the cross-level moderating effect of leader support were significant, and the moderating effect of job autonomy was not significant. In particular, the positive effect of daily job crafting on daily job satisfaction was strengthened for members with low leader support. These findings highlight that leader support is an important social context in job crafting, and provides insights when members can get more advantages from their daily job crafting.

## 1. Introduction

As the business environment has changed rapidly, job crafting has received attention from many researchers in the organizational behavior field in a way that increases the member’s meaning of the work [1]. Job crafting is defined as “the physical and cognitive changes individuals make in the task or relational boundaries of their work” [2]. In particular, recent studies have addressed job crafting as a daily behavior which members take the initiative in satisfying through their own motivation since the work cycle has become more short-term than in the past [3,4]. Empirical studies of daily job crafting primarily explored antecedents and daily job crafting’s effects, particularly mechanisms that increase the daily work engagement and performance of the members [5,6,7]. Despite the growing flow of job crafting researches, there is still a lack of understanding concerning the boundaries under which job crafting works [8,9].

Many contexts were not well constructed to fulfill intrinsic motivations and provide sufficient resources to members [10]. In addition to this, given that job crafting can serve as a strategy for these contexts, research to identify which members are more effectively affected by the influence of daily job crafting on daily job satisfaction will be worthwhile. In this regard, we investigate the moderating effect of job autonomy and leader support as the two aspects of work context, which are job context and relational context respectively [11]. To be specific, since job crafting is a self-regulating behavior of finding the meaning of work intuitively, the effects of job crafting will depend on the context [12,13].

We suggest that the positive effect of the daily job crafting will be strengthened in insufficient situations where members’ needs or motivations are not met. This is because, if the work context is deficient to fulfill individuals’ own values, individuals show a negative attitude and behavior by questioning the meaning and identity of their work [14,15]. In real workplaces, as individuals perform different jobs and also receive a different amount of support, not all individuals are fully motivated to work [16]. Individuals with low job autonomy have to work in a set way, so they can doubt what value their work activities create [17]. In addition, members who do not receive support from the leader will find it difficult to achieve their goals and enhance themselves by performing their job [18]. For these individuals, job crafting will serve as an act to discover the meaning of their work, and thus make them more positively evaluate their work [19]. Hence, by filling in the insufficient factors of the context, daily job crafting will more effectively inhibit negative reactions and work to ensure daily satisfaction with the job.

The purposes of our research are as follows. First, we examine the positive effect of daily job crafting on daily job satisfaction. Next, we aim to identify the moderating effect of job autonomy and leader support on this relationship to demonstrate that daily job crafting works more effectively for individuals with insufficient motivation. Our study contributes to the job crafting field by highlighting the role of daily job crafting in the daily satisfaction of a job. In particular, we demonstrate how the effects of job crafting vary depending on the characteristics of the job and the degree of support, and which members can obtain the advantage of job crafting more effectively.

## 2. Theoretical Backgrounds and Hypotheses

### 2.1. Daily Job Crafting and Daily Job Satisfaction

In the last few years, there has been a growing interest in research on daily job crafting [5,7]. Due to constant change in the work environment, the task-management cycle of the members itself are becoming shorter and it has become important to plan and carry out work-related goals every day [9]. Daily job crafting can be described by the explanation of Tims, Bakker, and Derks [20], who use the job demands-resources (JD-R) model to elaborately account for job crafting behaviors performed in a daily work environment. To empirically capture the actual crafting behaviors that increase the individual’s meaning and motivation of the work emphasized by Wrzesniewski and Dutton [2], they used the concepts of job demand and job resource [13,21]. Job demand refers to the job aspects that require sustained efforts, while job resource refers to the job aspects that enable job demands, personal development, and job goals to be achieved [22].

Job satisfaction is a key attitude variable that represents a member’s positive attitude toward their organization [23]. It has also been discussed at the within-person level lately; daily job satisfaction refers to a member’s assessment of the daily job and daily job experience [24]. We expect that daily job crafting will have a positive relationship with the daily job satisfaction. This is because, by regulating the meaning of one’s work intuitively, daily job crafting increases an individual’s motivation for their job and enables them to judge their job more positively [1,12,13]. Moreover, individuals can satisfy their needs and motivations through job crafting, specifically by developing their job abilities with more challenging jobs to form a sense of self-enhancement or self-esteem [25]. For example, crafters are likely to experience successful goal achievements by regulating their job into goals that are more available to accomplish [20]. Individuals can also expand the relational boundary by contacting someone who has not been in touch and thereby increasing social support [2]. Therefore, with increased motivation and a positive workplace experience through job crafting, individuals will evaluate the job of a particular day more satisfactorily. Based on the above discussions, we propose the following hypothesis:

**Hypothesis** **1.** *Daily job crafting has a positive relationship with daily job satisfaction.*

### 2.2. The Moderating Role of Job Autonomy

Depending on the work context, an individual’s motivation varies as per their job’s characteristics, which makes the effect of job crafting different [11]. Job autonomy, one of the representative job characteristics that are defined as an individual’s perception of the degree of freedom in handling the job and making a job-related decision [17], has a similar feature to job crafting, in that it increases an individual’s motivation by empowering individuals to regulate their jobs. Therefore, this study predicts that although daily job crafting increases daily job satisfaction, this advantage of job crafting will be enhanced for members with lower job autonomy, who are not motivated by their job context.

Members have a low job autonomy less choice in determining and implementing their work methods [17]. For example, they have to work on a set schedule and cannot choose their own way of working either. Under this context, the designed job is not sufficient to motivate members; therefore, they will consider their own strategic behaviors such as job crafting a more effective way to cope with the situation [26]. In other words, it is hard for them to find meaning in their work based on the designed job, and thus their motivation for the job is more relatively up to their own regulating behavior [27,28]. Since job crafting is considered as a key factor to seek the meaning of work, these members will be more likely to get benefits from their job crafting behavior which fulfills intrinsic motivations. However, members with highly autonomous jobs achieve a higher level of motivation from their given job condition and accordingly evaluate their job positively [26]. Therefore, we propose the following hypothesis:

**Hypothesis** **2.** *The positive effect of daily job crafting on daily job satisfaction will be strengthened when job autonomy is low more than when it is high.*

### 2.3. The Moderating Role of Leader Support

Along with the job context, the relational context also serves as an important factor in determining how job crafting affects members [9]. This study considers leader support as a crucial relational condition in the workplace which represents the leader’s series of helping for the member like providing job resources (i.e., information) [29]. Members with low leader support find it difficult to be inspired by their work and to develop the skills and resources necessary to achieve their goals since the work-related information and feedback on their work is not provided enough [18]. If the work’s worth is not fulfilled by the leader, members go the extra mile to find meaning in their work and will focus strongly on their own regulating behaviors [20,21]. In other words, when the available resources from the leader are insufficient, the members will secure resources to achieve their daily jobs satisfactorily through activities that seek meaning on their own or enlarge their boundaries [19]. For example, members can manage this insufficient situation by seeking valuable information to develop their capabilities or by expanding their social boundaries to get aids from another source or from infrequent connections.

Therefore, for members with low leader support, the effect of job crafting will be more significant because this behavior is considered to be a more effective strategy in shaping their work motivation. In other words, job crafting behavior complements an insufficient motivation for members to successfully overcome a situation where there is not enough support so that they can achieve daily job satisfaction. In contrast, members with a high level of leader support successfully establish the meaning of their work, and so they experience daily job satisfaction. We thus derive the following hypothesis:

**Hypothesis** **3.** *The positive effect of daily job crafting on daily job satisfaction will be strengthened when the leader support is low more than when it is high.*

**Figure 1:**
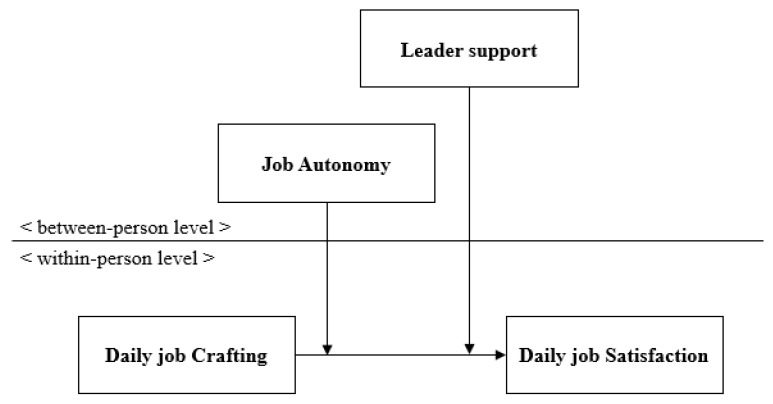
Proposed research model.

Figure 1 displays our research model.

## 3. Methods

### 3.1. Sample and Procedure

We collected our data through experience sample methods (ESM) to accomplish the objectives of this study and collected 10 consecutive workdays from each participant (2 weeks). The ESM method is recommended as a useful way to capture a member’s daily workplace experiences by repeatedly asking participant responses on a daily or weekly basis [30]. Since ESM could capture employee’s daily experiences more accurately by reducing the bias that arises from just recalling memories [31], we regarded it as an appropriate data collection method to verify the effect of daily job crafting.

However, the ESM requires the repeated collection of responses, so there is a potential problem that may result in a selective non-response [32]. To reduce this problem, we met with managers and participants in advance to explain the purpose of the survey and explained that it took ten days to complete. If an employee agreed to participate, they were asked to answer the paper-and-pencil survey questionnaires. We provided monetary compensation to induce participation and also sent messages to encourage participation every three days. In addition to this, we notified that the survey should be conducted in the afternoon and asked participants to record the response time at the bottom of the questionnaire.

A total of 143 employees from various organizations in South Korea were invited to our survey and we finally collected responses from 121 employees (response rate was 84.62%). The average age of the participants was 32 years (sd. = 7.91), with 61.9% of them being male, and 89.4% having a bachelor’s degree or above. The organization’s industry included 47.4% in manufacturing, 10.5% in finance, 0.9% in distribution, 15.8% in service, and 25.4% in others. Regarding the type of job, 71.7% were office workers, 12.4% were in sales, 7.1% were in a technical post, and 8.9% were in another area. The average tenure of participants was 71 months (sd. = 85.19).

### 3.2. Measures

Before starting the daily survey, participants were asked to report the between-person level variables which were demographic variables (gender, age, organization, tenure, job, and position), job autonomy, and leader support. The daily survey measured the level of job crafting and job satisfaction every day. All measures used a 5-point Likert-type scale ranging from (1) “strongly disagree”, to (5) “strongly agree”.

#### 3.2.1. Within-Person Level

***Daily job crafting*.** To measure job crafting, we used a modified version of a job crafting scale by Petrou and his colleagues [7]. The day-level job crafting questionnaire consisted of three subscales: seeking resources, seeking challenges, and reducing demands. Sample items were “Today, I have asked others for feedback on my job performance,” “Today, I have asked more tasks if I finish my work.” and “Today, I have tried to ensure that my work is emotionally less intense.” The Cronbach’s alpha was 0.74.

***Daily job satisfaction*.** Daily job satisfaction was measured with 3 items. To measure daily job satisfaction, we used a modified version of daily job satisfaction by Loi, Yang, and Diefendorff [33]. A sample item was “At present, I am satisfied with my job.” The Cronbach’s alpha was 0.88.

#### 3.2.2. Between-Person Level

***Job autonomy*.** We measured job autonomy using the 3 items developed by Morgeson and Humphrey [34]. Sample items include “The job allows me to make my own decisions about how to schedule my work”,“The job allows me to make a lot of decisions on my own”, “The job allows me to make decisions about what methods I use to complete my work.”. The Cronbach’s alpha was 0.82.

***Leader support*.** Based on the recommendation from Eisenberger and his colleagues [29], we utilized 8 items developed by Eisenberger, Huntington, Hutchison, and Sowa [35] to measure leader support. Sample items include “My leader is willing to extend himself/herself in order to help me perform my job to the best of my ability” and “My leader is willing to help me when I need a special favor”. The Cronbach’s alpha was 0.94.

***Control variables*.** To enhance the validity of our study, we included the following control variables in the analysis. We used individuals’ gender, age, organization, tenure, job and position as control variables. In particular, the organization and job were used to control potential effects, since job autonomy can vary from industry to industry or from job to job. Gender was a categorical variable with 1 as male and 0 as female. We created a dummy variable for the organization (i.e., 1 as the manufacturing industry and 0 as the others), job (i.e., 1 as office workers and 0 as the others), and position (i.e., 1 as staff and 0 as the others). Furthermore, we controlled the between-person level job crafting by using the average value of daily job crafting.

### 3.3. Variance Partitioning

Before examining our hypotheses, we conducted null model analyses to ensure that reasonable variances are distributed at the within-person level and calculate the ratio. For daily job crafting, 52% of the variances were at the within-person level. Daily job satisfaction also included substantial variances at the within-person level, and the ratio was 68%. Consequently, significant amounts of variance are left to be explained by within-person variations, justifying the within-person level approach and the use of Hierarchical Linear Modeling (HLM; i.e., variance at within-person and between-person levels).

## 4. Results

### 4.1. Confirmatory Factor Analysis

Table 1. presents the confirmatory factor analysis (CFA) results. We conducted CFA on four major variables including daily job crafting, daily job satisfaction, job autonomy, and leader support. Root mean square error of approximation (*RMSEA*), comparative fit index (*CFI*), and Turker-Lewis index (*TLI*) were used as indices to indicate the validity of the model. According to Muliak and colleague’s [36] and Gierl and Rogers’s [37] recommendation, the model is reasonable when *RMSEA* < 0.08, *CFI* > 0.90, and *TLI* > 0.90. Based on this cutoff criterion, our proposed four-factor model (χ^2^ (161) = 1052.03, *RMSEA* = 0.07, *CFI* = 0.93, *TLI* = 0.91) has been found to be suitable for our data and also relatively presented a better fit than other alternative models: model 2 (combined daily job crafting with daily job satisfaction as one factor; χ^2^ (164) = 1650.74, *RMSEA* = 0.09, *CFI* = 0.88, *TLI* = 0.85); model 3 (additionally combined job autonomy with leader support as one factor; χ^2^(166) = 2654.62, *RMSEA* = 0.11, *CFI* = 0.80, *TLI* = 0.75); and model 4 (combined all variables as one factor; χ^2^(167) = 4907.71, *RMSEA* = 0.15, *CFI* = 0.63, *TLI* = 0.53).

### 4.2. Hypotheses Testing

Table 2. presented the means and standard deviations for all variables. In the within-person level, daily job crafting was significantly and positively correlated with daily job satisfaction (r = 0.45, *p* < 0.01).

We used hierarchical linear modeling (HLM) to test our hypotheses. We conducted group mean centering at the within-person level (level 1), and grand mean centering at the between-person level (level 2). First, we tested a null model in which no predictors were entered. Next, we included the control variables (step 1), main predictors (step 2), and cross-level interaction terms (step 3). Table 3 summarizes HLM results. Hypothesis 1 predicted that daily job crafting would affect daily job satisfaction. As shown by the results in Table 3, model 2, daily job crafting was positively related to daily job satisfaction (γ = 0.64, *p* < 0.01). Hence, hypothesis 1 was supported.

Hypothesis 2 predicted job autonomy would moderate the relationship between daily job crafting and daily job satisfaction negatively. The interaction term, however, was not significant (γ = 0.11, *p* < n.s.), and thus, hypothesis 2 was rejected.

**Figure 2:**
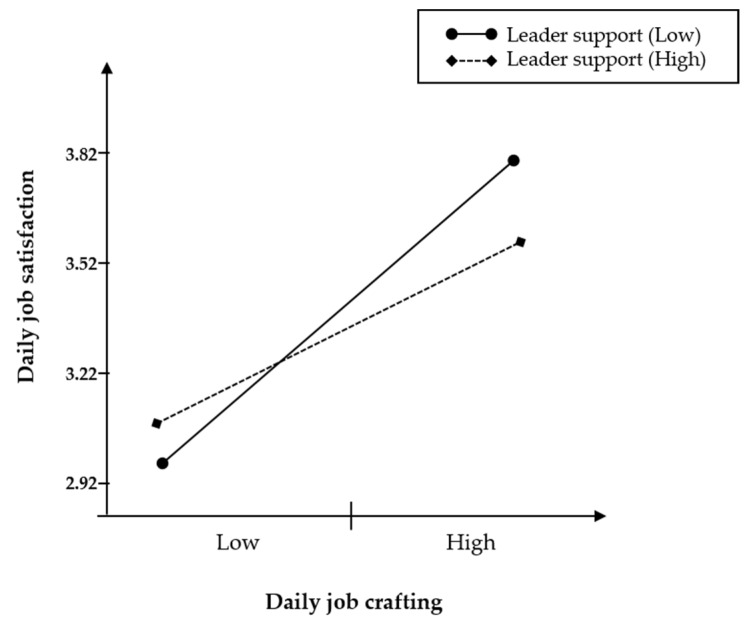
Cross-level moderating effect of daily job crafting and leader support on daily job satisfaction.

Hypothesis 3 predicted that leader support would moderate the relationship between daily job crafting and daily job satisfaction. We predicted when leader support is low, the relations would be strengthened. As shown by the result in model 3 of Table 3, the cross-level interaction term was significant (γ = −0.17, *p* < 0.05). We present this interaction graphically in Figure 2. Thus, hypothesis 3 was supported.

## 5. Discussion

The purpose of this study was to reveal the effect of daily job crafting on daily job satisfaction and the moderating effect of job autonomy and leader support on this relationship. As we predicted, daily job crafting had a significant positive effect on daily job satisfaction. Although the moderating effect of job autonomy was not statistically meaningful, leader support was found to have significantly moderated this relationship. Specifically, it was demonstrated that the positive effect of daily job crafting on daily job satisfaction was strengthened for those members with low leader support more than those with high leader support. These findings provide the following described implications, and the rejected moderating effect was as it is discussed in detail below.

### 5.1. Theoretical Implications

First, from a theoretical perspective, our research contributes to the job crafting field by highlighting the role of daily job crafting as an antecedent of daily job satisfaction. Recent meta- research into job crafting emphasized that as job crafting is conceptualized at the within-person level, the effect of job crafting also needs to be considered at the within-person level [1,9]. In response to these proposals, our results are empirical findings that reveal job crafting can affect job satisfaction at the within-person level. In addition, these results are also in the same line with those of daily studies focusing primarily on the relationship with work engagement and those that reveal the relationship between job crafting and job satisfaction at the between-person level [3,7,25], but are different and thus extend the understanding of job crafting. Moreover, it adds to the significance that it makes clear the effects of daily job crafting in real-world workplaces using the ESM method.

Second, we found in this study that under relatively low leader support conditions, the positive relationship between daily job crafting and daily job satisfaction was further strengthened. This may indicate that if the leader lacks support, it can be a member’s effective behavioral strategy to utilize the benefits of daily job crafting for their daily job satisfaction. This is because, while previous studies looked at which circumstances could enhance the effectiveness of job crafting primarily [25,38], we demonstrated that even in the context of deficiencies, the effect of daily job crafting can be strengthened. Moreover, this is in line with Wang, Demerouti, Blanc, and Lu [39] found in a recent study that suggests that the advantages of job crafting will be more fortified during tough times, but this study further implies that job crafting can be an effective self-regulating behavior to manage daily motives and attitudes by capturing these phenomena at the within-person level as well. Therefore, it can be interpreted that our result is due to the nature of the job crafting, based on which we add the importance and value of job crafting to existing job crafting research flows.

Third, previous studies had treated job design as an important issue and intended to motivate members and achieve performance through supplementing job characteristics [17,27]. On this basis, we explained our hypothesis logically, but contrary to our expectation, only the main effect of daily job crafting was a signification and the moderating effect of job autonomy was not. It can be interpreted that daily job crafting plays a crucial role in members’ experience of daily job satisfaction, and thus the main effect of daily job crafting has overridden the moderating effect. In other words, no matter how well the organizations improve members’ job context, members have to engage in crafting behavior to experience daily job satisfaction. For this reason, it may be considered that the hypothesis has been rejected and that no moderating effect has occurred.

### 5.2. Practical Implications

Along with theoretical significances, the practical implications are as follows. First, providing satisfactory and suitable conditions for every member of the organization is a real challenge. Perhaps, this argument may be ideal at the organizational level, particularly considering the fact that the needs of individual members are more diverse and the demands of the work environment change. However, based on the results of this study, members can perform their daily job satisfactorily by crafting their own daily jobs even if the leader does not provide adequate support. Therefore, daily job crafting can serve as an appropriate alternative to complement organizational aids or practices that can form a member’s daily work motivation. Hence, we suggest to practitioners that it is important to establish an environment in which members can craft their job easily and managers need to encourage their members to do so.

In addition, organizations should inform their members of these benefits of job crafting and train them to take advantage of it. Although daily job crafting is beneficial to members themselves, they may be reluctant to do so because it has some aspect of uncertainty and consumes their job resources [1]. Thus, along with building a culture that encourages crafting, it is important to educate members about how they can actually craft and how this can be helpful to meet their needs and motivations. Training programs or coaching through leaders might be one way, and this will allow effective management of members to move on to enhancing organizational effectiveness.

### 5.3. Limitations and Future Research Directions

In spite of these implications, we recognize several of the following limitations. First, we took into account the between-personal level moderators by placing our attention on cross-level moderating effects, but future studies can also consider within-person level moderators. For example, Sonnentag, Mojza, Demerouti, and Bakker’s [40] study addressed the within-person level work-related moderator in the relationship between daily work engagement and daily recovery, and we also suggest that the characteristics of the within-person level may affect daily job crafting results. This will increase the understanding of job crafting’s boundary condition.

Second, since all of our variables were measured at the same time by the members, potential common method variance issues can be raised [41]. Although we conducted group-mean centering and controlled the individual means to exclude the explanation through individual differences, we acknowledge that the potential problem was not completely overcome [40]. Thus, in future studies, leader support can be measured by the leader. Furthermore, by the spacing between the measurement points of daily job crafting and daily job satisfaction, the role and effect of daily job crafting as an antecedent will be more clearly illuminated.

Third, it is worthwhile to shed a light on the effect of daily job crafting on the daily job attitude of members, but it is regrettable that the effect on member’s performance has not been addressed. Recently it has been shown that job crafting has a positive effect on performance at the between-person level [42], and daily job crafting can also be linked to performance. More elaborately, it is possible to measure performance that can be measured by the leader to reduce potential bias and produce clearer research results.

Finally, in Parker, Wang, and Liao’s [43] recent review dealing with the moderators of the relationship between proactive behavior and outcomes, the boundary condition is classified into three categories: task, social, and self-related. Although we dealt with job autonomy as a task aspect and the leader support as a social aspect of work context, factors related to the self could also be considered. For example, the self-regulatory focus could be addressed as one of the individual characteristics. Therefore, we expect to increase our understanding of job crafting through considering the boundary categories and the level of analysis discussed above in future research.

## 6. Conclusions

We expanded the job crafting field, revealing the moderating effect of job autonomy and leader support. Specifically, our findings reveal that the benefits of daily job crafting are more effectively represented in an insufficient environment (low job autonomy or low leader support). This suggests that when situations make it difficult to meet the needs and motivations of members, the daily initiative behaviors enhancing the meaning of work can function effectively to form satisfaction with a job. Although we have focused on the job and social aspects of the work context, we expect to broaden existing understanding of the effects of job crafting in consideration of the additional boundary conditions.

## Figures and Tables

**Table 1:** Results of confirmatory factor analysis.

Model	Factors	χ^2^	*df*	*RMSEA*	*CFI*	*TLI*
1	Four factors model	1052.03	161	0.07	0.93	0.91
2	Three factors model (combined daily job crafting with daily job satisfaction)	1650.74	164	0.09	0.88	0.85
3	Two factors model	2654.62	166	0.11	0.80	0.75
4	One factor model	4907.71	167	0.15	0.63	0.53

**Table 2:** Means, standard deviations, and correlations.

Variables	Mean		SD		1	2	3	4	5	6	7	8	9	10
*Between-person level*														
1. Gender ^a^		0.62		0.49										
2. Age	32	0.46	7	0.91	0.09									
3. Organization ^b^		0.47		0.50	0.19 *	0.07								
4. Tenure ^c^	70	0.94	85	0.19	0.07	0.75 **	0.16							
5. Job ^d^		0.72		0.45	0.03	0.01	0.07	0.09						
6. Position ^e^		0.71		0.46	0.10	0.69 **	0.02	0.58 **	0.04					
7. Job autonomy	3	0.58		0.74	0.10	0.39 **	0.04	0.29 **	0.04	0.36 **	(0.82)			
8. Leader’s support	3	0.43		0.82	0.15	0.24 *	0.01	0.09	0.04	0.18	0.21 *	(0.94)		
*Within-person level*														
9. Daily job crafting	3	0.21		0.60									(0.74)	
10. Daily job satisfaction	3	0.17		0.81									0.45 **	(0.88)

**Note**. *N* = 121 (between-person level); 988 (within-person level). Reliabilities are reported on the diagonal. ^a^ Male = 1, female = 0; ^b^ manufacturing industry = 1, the others = 0; ^c^ scale is month; ^d^ office workers = 1, the others = 0; ^e^ staff = 1, the others = 0. * *p* < 0.05, ** *p* < 0.01.

**Table 3:** HLM Regression: Dependent variable is daily job satisfaction.

Variables	Model 1			Model 2			Model 3		
Intercept	3	0.13 **	(0.20)	3	0.31 **	(0.18)	3	0.30 **	(0.18)
*Control*									
Gender		0.13	(0.12)	-	0.05	(0.11)	-	0.05	(0.11)
Age		0.02	(0.01)		0.01	(0.01)		0.01	(0.01)
Organization	-	0.26 *	(0.12)	-	0.26 *	(0.10)	-	0.26 *	(0.10)
Tenure	-	0.00	(0.00)	-	0.00	(0.00)	-	0.00	(0.00)
Job		0.05	(0.13)		0.04	(0.11)		0.04	(0.11)
Position		0.13	(0.19)		0.05	(0.16)		0.06	(0.16)
*Within-person level*									
Daily job crafting					0.64 **	(0.06)		0.62 **	(0.06)
Between-person level									
Job crafting (mean)					0.68 **	(0.11)		0.68 **	(0.11)
Job autonomy					0.15 *	(0.07)		0.14 *	(0.07)
Leader support				-	0.01	(0.06)		0.01	(0.06)
Cross-level interaction									
Daily job crafting × Job autonomy								0.11	(0.08)
Daily job crafting × Leader support							-	0.17 *	(0.08)

**Note:** * *p* < 0.05, ** *p* < 0. 01. Numbers outside parentheses are the coefficient, and numbers in parentheses are the standard error.
